# Efficacy and Tolerability of Two Different Low-Volume Split-Dose Polyethylene Glycol Electrolytes Solution Bowel Preparation for Morning Colonoscopy

**DOI:** 10.1155/2022/8169649

**Published:** 2022-08-31

**Authors:** Hefeng Tian, Hui Li, Xuanrui Zhu, Wenlong Liu, Ying Fan, Lei Shi, Xiu Wang

**Affiliations:** The First Hospital of Jilin University, Changchun, Jilin, China

## Abstract

**Methods:**

A total of 120 patients were randomized to receive either the control group (*n* = 64) or the experimental group (*n* = 65). Patients in the control group adopted the low-volume split-dose regimen one, and patients in the experimental group adopted the low-volume split-dose regimen two. Those randomized to regimen one were instructed to take 0.75 L PEG two hours after dinner the day before the colonoscopy and 1.5 L PEG 4 hours before the colonoscopy. Patients assigned to regimen two were invited to consume 1.5 L PEG two hours after dinner the day before the colonoscopy and 0.75 L PEG 4 hours before the colonoscopy. The quality of bowel preparation, rated according to a Boston Bowel Preparation Scale (BBPS), represented the primary outcome measure. Tolerability, satisfaction, and lesions detection rated were secondary outcomes.

**Results:**

There was no significant difference between the transverse colon and right colon scores between the two groups (*P* > 0.05). The low-volume split-dose regimen two showed a higher success rate for cleansing of the right colon and overall colon (*P* < 0.05). For the comparison of the patients' bowel tolerance, there were no statistical differences between the two groups regarding thirst, abdominal pain or abdominal discomfort, abdominal distension, dizziness or headache, anal discomfort, and sleep disturbance (*P* > 0.05). However, regimen two had significantly less nausea, vomiting, and fatigue than regimen one (24.62% vs. 42.19%, *P*=0.034; 10.77% vs. 25.00%, *P*=0.035; 6.15% vs. 21.88%, *P*=0.010, respectively). Patient-reported satisfaction and willingness to repeat the bowel preparation were significantly higher for low-volume split-dose regimen two than for low-volume split-dose regimen one (*P*=0.011; *P*=0.015).

**Conclusions:**

In early morning colonoscopies, the bowel-cleansing efficacy and patient tolerability of low-volume split-dose regimen two were superior to low-volume split-dose regimen one.

## 1. Introduction

In the latest global cancer statistics, colorectal cancer is the third most common cancer after prostate cancer and lung cancer [1–3]. According to Chinese cancer statistics, colorectal cancer ranks third and fifth in the incidence and death of malignancies, respectively. Colonoscopy as the only gold standard for the diagnosis and treatment of colorectal cancer and precancerous lesions, is closely associated with the “adenoma-cancer” pathway [4, 5]. Adenoma detection rate is not only a standard of colonoscopy performance but also a quality indicator specific to colonoscopy. Related studies have shown that the more adequate the bowel preparation, the higher the adenoma detection rate [6–8]. When the quality of bowel preparation is seriously unqualified, patients will be forced to give up diagnosis and treatment, make an appointment to take bowel-clearing drugs, and conduct colonoscopy again, which will increase the economic burden on patients and consume their time and energy [8, 9].

An ideal bowel-clearing drug is easily accepted by most patients, inexpensive, and can achieve high-quality bowel preparation [10–12]. The PEG solution is the preferred bowel-clearing drug recommended by the bowel preparation guidelines for digestive endoscopic diagnosis and treatment around the world. In European and American countries, it is generally recommended to take high-volume (≥3 L) PEG solution. Nevertheless, high-volume preparations usually have a high incidence of side effects and are poorly tolerated [10, 11, 13]. In contrast, the low-volume PEG solution is favored by the patients in terms of tolerability, compliance, and side effects such as abdominal distension, nausea, and vomiting [14–16]. Some studies have shown that a split-dose regimen for morning colonoscopy is more effective than nonsplit-dose regimen preparations [10, 17, 18]. In the split-dose regimen, some of the preparations were taken on the night before the colonoscopy and the remaining ones on the morning of the colonoscopy, which can better avoid contaminating the bowel wall again [19].

But so far, there are little data on low-volume split-dose PEG solution bowel preparation for morning colonoscopy. Therefore, the purpose of this study is to compare the efficacy and tolerability of two different low-volume split-dose PEG solution bowel preparation for morning colonoscopy based on domestic and foreign guidelines and combined with the clinical actual situation, explore the best PEG solution regimen and improve the effect of colonoscopy, patient compliance, and comfort.

## 2. Methods

### 2.1. Study Design

This is a prospective, randomized, controlled, endoscopic-blinded trial at the Endoscopy Center of the First Hospital of Jilin University, Changchun, China. The regimen was approved by the local ethical review committee and all participating subjects had signed informed consent for electronic colonoscopy. I and my co-authors had access to the study data and reviewed and approved the final manuscript.

### 2.2. Patients

Patients undergoing electronic colonoscopy from the outpatient department of the First Hospital of Jilin University from May 2021 to November 2021 were selected as the study subjects. Inclusion criteria: >18 years of age; hospital outpatient patients undergoing electronic colonoscopy; taking polyethylene glycol bowel-clearing drugs; willing to voluntarily participate in the study; communicating with them normally. Exclusion criteria: severe gastrointestinal obstruction, perforation, or bleeding; severe cardiopulmonary, liver, and kidney; poor bowel preparation that affects electronic colonoscopy.

### 2.3. Randomization and Intervention

Their recruited study subjects were randomly assigned to receive bowel preparation from the experimental and control groups according to a computer-generated list with a 1 : 1 allocation ratio. Randomization was performed in a 4-block size without informing the study team before the assignment. Patients with written consent will be assigned an opaque, sealed envelope that was required to follow the regimen in the envelope. Colonoscopy and assessment of bowel preparation quality were performed individually by the same experienced endoscopist, who was not aware of the experimental grouping and did not participate in bowel preparation-related activities.

Patients in the control group adopted the low-volume split-dose regimen one, and patients in the experimental group adopted the low-volume split-dose regimen two. Those randomized to regimen one were instructed to take 0.75 L PEG (6 bags of A agent and 6 bags of B agent) at the speed of 250 ml every 10 minutes two hours after dinner the day before the colonoscopy and 1.5 L PEG (12 bags of A agent and 12 bags of B agent) at the same speed 4 hours before the colonoscopy. Patients assigned to regimen two were invited to consume 1.5 L PEG (12 bags of A agent and 12 bags of B agent) every 10 minutes two hours after dinner the day before the colonoscopy and 0.75 L PEG (6 bags of A agent and 6 bags of B agent) at the same speed 4 hours before the colonoscopy. Patients were also asked to consume a semiliquid diet the night before the colonoscopy and were banned on the morning of the day of the procedure. On the morning of the examination, the patient information was accurately recorded by using face-to-face methods. Initially, the general demographic data of the patients were recorded during the interview. Then, the patients were asked about their tolerability, acceptability, and satisfaction with bowel preparation after taking the PEG solution. During the course of the colonoscopy, each patient's quality of bowel preparation was independently assessed by the same endoscopist who had worked in the endoscopy center for more than five years and had more than 2,000 colonoscopy cases per year. All patients completed colonoscopy during the 8:00 am to 10:00 am period on the day of the procedure.

### 2.4. Patient Information Collection

Under the guidance of the endoscopic experts, we developed a comprehensive and unified questionnaire of patient demographic information for electronic colonoscopy by reviewing the extensive norms and literature on bowel preparation around the world. It mainly includes the general demographic data of the study subjects: the serial number, age, weight, height, gender, body mass index (BMI), education (master and doctor, junior college and undergraduate, high school, junior high school, and below), history of the basic disease (diabetes, heart disease, hypertension), other diseases (thyroid surgery, radical resection, abdominal surgery, polypectomy) and indications for colonoscopy (diarrhea, abdominal pain, abdominal distension, physical examination, stool habit change).

### 2.5. Outcome Measures

#### 2.5.1. Primary Outcome

The primary outcome measure was patient bowel cleansing. The efficacy of bowel preparation in both groups was evaluated by using the validated Boston Bowel Preparation Scale (BBPS) [20]. The endoscopist scored the ascending, mid (transverse and descending), and rectosigmoid segments of the colon separately. Each segment was evaluated as follows: excellent: 3 (the bowel mucosa was well observed, with basically no residual feces, turbid fluid, and stains), good: 2 (the bowel mucosa was well observed, but a small number of feces, turbid fluid and stains remained), poor: 1 (due to the residual feces, turbid liquid and stains, some mucosa cannot be observed), inadequate: 0 (due to the residual feces, turbid liquid and stains, the whole section of the mucosa cannot be observed) according to the difference of bowel preparation quality. The highest total score was 9 and the lowest total score is 0. If the total score is not less than 6 and each segment score is not less than 2, the patient has adequate bowel preparation, and if the total score is less than 6, the patient has poor bowel preparation. If the patient's bowel preparation quality is so poor that the endoscopy cannot reach the colon segment, the segment will be automatically rated as inadequate by the endoscopist. At the time of colonoscopy, the endoscopist can obtain a poster-size image and standard scoring examples for reference.

#### 2.5.2. Secondary Outcomes

The secondary outcome measure included tolerability of bowel preparation, satisfaction with bowel preparation, and lesions detection.

### 2.6. Tolerability

Before the colonoscopy, the investigators used the patient bowel preparation tolerability questionnaire to evaluate the tolerability of bowel preparation in the two groups [21]. Then the investigators asked each patient who had completed bowel preparation and recorded each item in the questionnaire to analyze whether the incidence of tolerability to the two different low-volume split-dose regimens varied and further analyzed the relationship between the incidence of tolerability and bowel cleansing in the two groups. The content of the tolerability questionnaire mainly included thirst, abdominal pain or abdominal discomfort, abdominal distension, nausea, vomiting, dizziness or headache, fatigue, anal discomfort, sleep disorders, etc. Each item is divided into severe symptoms, moderate symptoms, mild symptoms, and no symptoms. The more the number of asymptomatic patients, the better the tolerability.

### 2.7. Satisfaction

Before the colonoscopy, the investigators used the patient bowel preparation satisfaction questionnaire to evaluate the satisfaction of bowel preparation in the two groups [22]. Then the investigators asked each patient who had completed bowel preparation and recorded each item in the questionnaire to analyze whether the incidence of satisfaction with the two different low-volume split-dose regimens varied and further analyzed the relationship between the incidence of satisfaction and bowel cleansing in the two groups. The content of the satisfaction questionnaire mainly includes easy or difficult-to-consume: the study drug, the dose of the drug, the taste of the drug, the flavor of the drug, overall satisfaction with the regimen, and willingness to repeat bowel preparation. Each item is divided into five levels: very unsatisfactory, unsatisfactory, general, satisfactory, and very satisfactory. The more the proportion of patients who are satisfied, the higher the satisfaction degree is.

### 2.8. Lesions Detection

During the colonoscopy, the detection of bowel lesions (adenoma, polyps, inflammation, suspected cancer, etc.) was independently evaluated by the same endoscopist. The researchers made records to ensure the accuracy of the data, calculated the detection rate of bowel lesions, analyzed whether there was any difference in the detection rate of bowel lesions, and further analyzed the relationship between the detection rate of bowel lesions and bowel cleansing in the two groups.

### 2.9. Statistical Analysis

The sample size of this study assumes the same efficacy as the two low-volume split-dose regimens. Related published studies have shown that patients should achieve 80% successful cleansing rates. The difference in efficacy between the two low-volume split-dose regimens was 20%, which is considered to be clinically relevant. It was calculated that the initial sample size of 64 patients would be sufficient to reveal the set-up treatment effect difference of 20%, a probability of type I error of 0.05, efficacy of 80%, and effective value of 0.5. Seventy-one patients accounted for ∼10% of withdrawals.

All the data available in this study were processed by statistical product and service solutions (SPSS) 24.0 statistical software. Count data included general data of patients (gender, education, basic disease history, other diseases, indications of colonoscopy), adverse reactions, and bowel preparation satisfaction. We analyzed the count data using the Chi-square test, and the results were expressed using frequency (*n*) and percentage (%). If the theoretical frequency was less than 5, we would use the fisher exact probability method. The measurement data included the general data of colonoscopy patients (age, height, weight, BMI) and the scores of bowel cleansing effect. After the test and analysis, the study data conform to the normal distribution. We analyzed the measurement data using the Student's *t*-test, and the results were expressed using mean and standard deviation. All statistical tests were two-sided, and a *P* value less than 0.05 was considered statistically significant.

## 3. Results

**Figure 1:**
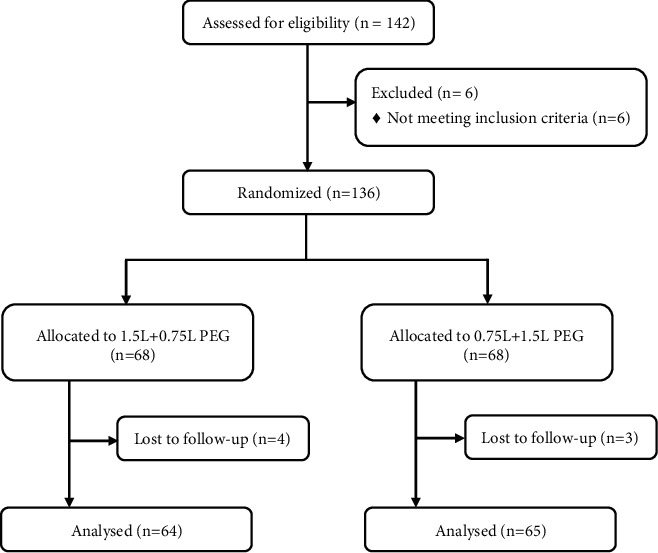
Flow diagram of the study.

From May to November 2021, 142 patients undergoing colonoscopy were recruited for this study, and six patients did not meet the inclusion and exclusion criteria. 136 patients were randomly assigned and distributed equally across the control group (0.75 L + 1.5 L) and experimental group (1.5 L + 0.75 L). A total of 4 patients in the control group canceled colonoscopy before bowel preparation, among which two patients canceled colonoscopy due to temporary work tasks, one patient asked to do colonoscopy in the afternoon, and one patient gave up colonoscopy. Finally, 64 patients in the control group were included in the data statistical analysis. A total of 3 patients in the experimental group canceled colonoscopy before bowel preparation, among which one patient canceled colonoscopy due to temporary work tasks, and two patients required colonoscopy in the afternoon. Finally, 65 patients in the experimental group were included in the data statistical analysis (Figure 1). The difference between the demographic data and the clinical characteristics of the two groups was not statistically significant and was comparable (*P* > 0.05). The specific data statistical results are shown in Table 1.

### 3.1. Bowel Cleansing

In terms of the rectum and sigmoid colon and overall colon score, the score of the experimental group (1.5 L + 0.75 L) was significantly better than that of the control group (0.75 L + 1.5 L) (2.54 ± 0.56 vs. 2.23 ± 0.70, *P*=0.006; 6.98 ± 1.02 vs. 6.34 ± 1.88, *P*=0.017). In terms of the right colon and transverse colon scores, there was no difference in scores between the 1.5 L + 0.75 L and 0.75 L + 1.5 L groups (2.12 ± 0.41 vs. 1.94 ± 0.68, *P*=0.071; 2.54 ± 0.56 vs. 2.23 ± 0.70, *P*=0.172), but the mean scores for the 1.5 L + 0.75 L group were relatively high. The 0.75 L + 1.5 L group of 52 patients had adequate bowel preparation, accounting for 81.25%, and the 1.5 L + 0.75 L group of 62 patients had adequate bowel preparation, accounting for 95.38%. Statistical analysis indicated a significant difference in adequate bowel preparation between the two groups (*P*=0.012). The specific data and statistical results are shown in Table 2.

### 3.2. Tolerability

Overall, the 1.5 L + 0.75 L group was better tolerated when compared to the 0.75 L + 1.5 L group.  Table 3 showed the results of tolerability. Thirst, abdominal pain or abdominal discomfort, abdominal distension, dizziness or headache, anal discomfort, and sleep disturbance were the main symptoms reported. There was no significant difference in the incidence rate between the 0.75 L + 1.5 L group and the 1.5 L + 0.75 L group. However, the results showed that patients using the 1.5 L + 0.75 L group regimen had significantly less nausea, vomiting, and fatigue than the 0.75 L + 1.5 L group (24.62% vs. 42.19%, *P*=0.034; 10.77% vs. 25.00%, *P*=0.035; 6.15% vs. 21.88%, *P*=0.010, respectively).

### 3.3. Satisfaction

After taking the bowel-clearing drugs through the comparative analysis of the data, the *P* values of easy or difficult to consume the study drug, the dose of the drug, the taste of the drug, the flavor of the drug were 0.826, 0.822, 0.805, and 0.603 between the two groups, respectively, which were not statistically significant. A greater proportion of patients in the 1.5 L + 0.75 L group had a high level of satisfaction and stronger willingness to repeat the bowel preparation compared with patients receiving 0.75 L + 1.5 L PEG (*P*=0.011 and *P*=0.015). The specific data and statistical results are shown in Supplementary Table 1.

### 3.4. Lesions Detection

In this study, the detection of bowel lesions in colonoscopy mainly included polyps, adenoma, inflammation, suspected cancer, or other lesions. Analysis of the lesions detection rate in both groups showed that *P* values were 0.530, 0.365, 0.668, 0.496, and 1.000, with no statistical difference (*P* > 0.05). The specific data and statistical results are shown in Supplementary Table 2.

### 3.5. Correlation Analysis

Correlation analysis was performed separately on the relationship between adequate bowel preparation and lesions detection rate in the 1.5 L + 0.75 L and 0.75 L + 1.5 L groups, which showed no significant difference (1.5 L + 0.75 L groups: *P*=0.602; 0.75 L + 1.5 L groups: *P*=0.202). The specific data and statistical results are shown in Supplementary Table 3. Correlation analysis was performed separately on the relationship between adequate bowel preparation and incidence of adverse reactions in the 1.5 L + 0.75 L and 0.75 L + 1.5 L groups, which showed no significant difference (1.5 L + 0.75 L groups: *P*=0.284; 0.75 L + 1.5 L groups: *P*=1.000). The specific data and statistical results are shown in Supplementary Table 4.

## 4. Discussion

Currently, colonoscopy is still the safest and most effective gold standard in the diagnosis and treatment of bowel diseases, and it plays an important role in the screening of colorectal cancer. Moreover, one of the key factors affecting the screening, diagnosis, and treatment of colorectal cancer is the quality of bowel preparation, which is closely related to the effectiveness of colonoscopy. Adequate bowel preparation is essential to improve the quality of the colonoscopy. The choice of bowel preparation method depends on efficiency, safety, cost, patient risk and tolerability, time and dose of the study drug, etc. In a split-dose regimen, low or high-dose preparations may have different effects on patients undergoing colonoscopy in the morning. Low-volume preparation regimens are relatively easy for patients to ingest. They may benefit from the lower intensity preparation method compared with a high-dose preparation regimen. Thus, the 1.5 L (PM) + 0.75 L (AM) regimen was evaluated in this study. The present prospective, randomized, controlled trial found that 1.5 L (PM) + 0.75 L (AM) regimen was better than 0.75 L (PM) + 1.5 L (AM) in providing adequate bowel cleansing. However, both groups can achieve adequate bowel preparation of more than 80%. Patients in the 1.5 L (PM) + 0.75 L (AM) group had higher tolerance and compliance and were more willing to repeat bowel preparation. To our knowledge, this is the first evidence-based study focused on low-volume split-dose PEG solution bowel preparation for morning colonoscopy, with evidence that 1.5 L (PM) + 0.75 L (AM) regimen provides adequate bowel preparation in patients undergoing morning colonoscopy.

In this study, patients in control and experimental groups used the 0.75 L (PM) + 1.5 L (AM) regimen and 1.5 L (PM) + 0.75 L (AM) regimen for bowel preparation, respectively. The endoscopist used BBPS to score each segment of the bowel, and the total score of each segment exceeded 6 points for adequate bowel preparation. Our results showed that the proportion of patients who could complete the bowel preparation was 81.25% and 95.38%, which was statistically significant. Matro et al. [23] found that the whole bowel preparation was adequate in 92% in the AM-only group (the first 1 L dose seven hours before colonoscopy and the second 1 L four hours before the colonoscopy) versus 94% in the PM/AM group (the first 1 L at 6 pm the night before colonoscopy, and the second 1 L four hours before colonoscopy) with no significant difference, inconsistent with the findings of our study. The different reasons may be due to the total dose increase of 0.25 L PEG in this study. The experimental group took 0.75 L PEG in the morning and the proportion of adequate bowel preparation was 95.38%, which is much higher than the results in other retrospective studies [24, 25]. Notably, with regard to patients with inflammatory bowel disease (IBD), no statistically significant differences between the two groups were found in adequate bowel preparation. Although the sample size of IBD patients was limited, the proportion of high-quality bowel preparation was higher in the 1.5 L + 0.75 L group than in the 0.75 L + 1.5 L group (91.7% vs. 80%, *P*=0.571). High-quality bowel preparation has an important role in detecting IBD-associated neoplasia, especially in flat minute foci [26–28]. IBD patients mostly have the characteristics of chronic recurrence, easy carcinogenesis, and intestinal lesions, and their intestines will be carefully examined. Therefore, the quality of bowel preparation requirements will be higher. More recently, consistent literature data confirmed that high-quality bowel preparation is a key factor in endoscopy [29, 30]. In this study, the satisfaction rate of adequate bowel preparation in the 1.5 L + 0.75 L group was higher than in the 0.75 L + 1.5 L group, and we analyzed the reasons and found in clinical practice, although the patients in both groups completed bowel cleansing according to the corresponding bowel preparation regimen, and determined that their last stool was clear water, the patients in the 1.5 L + 0.75 L group tolerated higher, and the proportion of vomiting was less than that of the 0.75 L + 1.5 L group, so the bowel cleansing of the patients in the 1.5 L + 0.75 L group was better than that of the 0.75 L + 1.5 L group. This study also confirmed the importance of taking split-dose drugs before colonoscopy and showed that an appropriate reduction of the morning dose can improve bowel cleansing and partly prevent patients from making bowel preparation again, saving medical resources. Since small doses of drugs usually produce high levels of adequate bowel preparation, the study design we adopted is a randomized controlled trial. Compared with previous split-dose regimens, this study used low-volume split-dose drugs to avoid the defects of patients taking large amounts of bowel scavenger in the morning and optimize the quality of bowel preparation.

The success rate of bowel preparation in this study was higher than that of other related studies. From the analysis of bowel data in each part, it can be seen that the bowel cleansing effect of the two groups was the best in the left colon and the worst in the right colon. However, the bowel cleansing effect of the 1.5 L + 0.75 L group was better than that of the 0.75 L + 1.5 L group in terms of segmentation score and total score, obtaining the best bowel preparation, which was similar to the study results of Wudong. Wu Dong et al. took a split-dose regimen of 3 L PEG (2 L PEG on the night before colonoscopy and 1 L PEG four hours before colonoscopy) and found left colon was 2.6 ± 1.0 points, the transverse colon was 2.4 ± 0.8 points, the right colon was 2.2 ± 1.2 points, and total colon was 6.9 ± 3.1 points. There were significant differences compared with the control group. Ye Leping et al. took a similar approach and concluded that low-volumes PEG solutions were more well-tolerated than split-dose solutions. Nevertheless, the effectiveness of the split-dose solution was not specifically analyzed. Further high-quality clinical experimental studies should be conducted in the future.

This study compared the occurrence of adverse reactions (thirst, abdominal pain or abdominal discomfort, abdominal distension, dizziness or headache, anal discomfort, and sleep disturbance) in the two groups, and the *P* value was greater than 0.05 with no statistical difference. However, there were significant differences in the occurrence of nausea, vomiting, and fatigue, consistent with the Kotwal' et al. study [31]. Related studies have also pointed out that reducing the dose of PEG solution in the morning makes it easier to ingest and reduces the occurrence of nausea and vomiting without affecting colonoscopy. The experimental group in this study also reduced the traditional morning dose of 1.5 L PEG to 0.75 L PEG without changing the total dose, which greatly reduced the occurrence of nausea and vomiting in patients. It may be that the patient's stomach has been emptied early in the morning, and the large dose of PEG solution goes into the digestive tract, which directly expands the stomach and stimulates the receptors of the gastric floor and gastric body. The resulting impulses reach along the afferent and efferent fibers in the vagal nerve to the parietal cells of the gastric mucosa. The release of acetylcholine through the endings causes a large secretion of gastric acid and damages the gastric mucosa. Thus, gastric peristalsis weakens, gastric emptying delays, bowel tension increases, and reverse peristalsis occurs, resulting in the reflux of duodenal contents into the stomach. Finally, patients are most likely to experience nausea and vomiting. However, the patients in the experimental group took 1.5 L PEG the night before colonoscopy, and because the stomach was not completely emptied, it was highly tolerated. In the early morning, patients took 0.75 L PEG, and although the stomach was emptied, taking small doses of PEG solution caused less gastrointestinal irritation and less severe physical adverse stimulation. Nausea and vomiting are the most common adverse reactions to PEG solution. Reducing the occurrence of nausea and vomiting will inevitably reduce the overall incidence of adverse reactions. Because the patient has experienced a long period of fasting, coupled with a large amount of digestive juice discharged, the patient may show physical fatigue or even a hypoglycemic reaction. Therefore, when giving patient health education, we should explain to the patient in advance that mild nausea, vomiting, and fatigue are normal reactions, and we can properly control the speed of medication to avoid other adverse events; if the physical reaction is serious and cannot be tolerated, medication should be stopped to prevent other complications.

Due to the characteristics of the PEG solution itself, there was not much difference in terms of easy or difficult to consume the study drug, the dose of the drug, the taste of the drug, and the flavor of the drug, which is similar to that reported in previous studies [32]. Our study found that patients were more satisfied with the low-volume split-dose regimen in the experimental group and were more willing to repeat bowel preparation. To make this study more convincing, a questionnaire was designed to investigate the willingness of patients to change the existing bowel preparation regimen in the hospital, and the results showed that more than half of the patients wanted to change the regimen, perhaps mainly because the existing regimen is prone to adverse reactions, which affected the quality of life of patients. By analyzing the data of this study, there was no correlation between bowel cleansing effect and lesions detection rate and adverse effects in the two groups. However, our present study found that reducing the dose of drugs taken in the morning can improve the lesions detection rate, which may have great clinical practical significance. It is well known that digest and mucus in the digestive tract accumulate over time, which may hinder the detection of colonic lesions. At the same time, our study took part of the bowel-clearing drugs on the day of the colonoscopy, which may increase the detection of patient lesions and improve the effectiveness of colonoscopy.

There were several limitations in this study. First, we did not compare the efficacy between 2.25 L split-dose PEG and 2.25 L single-dose PEG. Our center has previously conducted a clinical trial to address this issue. We found that the single-dose PEG regimen was not applicable to our hospital. In addition, the latest domestic data show that a single-dose PEG regimen is easy to cause more adverse reactions in patients. In order not to cause unnecessary discomfort in patients, our study abandoned this regimen. Second, the factors affecting bowel cleansing are age, hypertension, diabetes, etc. Although the baseline of the two groups in this study was comparable, further study should explore whether the personalized volume of PEG based on individual's presence of factors that influence bowel cleansing would be superior. Third, for patients with inflammatory bowel disease, we used BBPS to evaluate the efficacy of the patient's bowel preparation but did not further evaluate disease severity with the Mayo Endoscopic Score (MES) and the Ulcerative Colitis Endoscopic Index (UCEIS), which are currently commonly used in clinical trials [33]. Last, this is a single-center study with limited sample size, and the selection of study subjects was limited to outpatients in the endoscopic center of the hospital. The study results are not representative of all populations, so multicenter studies should be conducted in the future to validate these experimental study results and make the study results generalizable.

In conclusion, this study found that patients receiving the experimental group regimen before the morning colonoscopy could achieve a good bowel preparation effect. Although both groups had similar adverse effects, patients receiving the experimental group regimen were more tolerant, more compliant, more comfortable, and more willing to repeat bowel preparation. This study regimen can improve patient comfort and tolerance and provide an evidence-based basis for future studies on patient bowel preparation for colonoscopy.

## Figures and Tables

**Table 1:** Patient demographic data and clinical characteristics for colonoscopy.

Parameter	0.75 L + 1.5 L group (*n* = 64)	1.5 L + 0.75 L group (*n* = 65)	*T/χ* ^*2*^	*P* value
Sex (*n*, %)			1.304	0.253
Male	30 (46.88)	37 (56.92)		
Female	34 (53.12)	28 (43.08)		

Age (Mean ± SD)	53.56 ± 12.5	51.80 ± 14.35	0.742	0.459
BMI (Mean ± SD)	23.76 ± 3.31	23.58 ± 3.44	0.303	0.763

Degree of education (*n*, %)			3.003	0.096
Master and doctor	3 (4.69)	5 (7.69)		
Specialist and undergraduate	22 (34.38)	30 (46.15)		
Senior middle school;	16 (25.00)	14 (21.54)		
Junior high school and below	23 (35.93)	16 (24.62)		

Indications for colonoscopy (*n*, %)				
Diarrhea			2.022	0.155
Yes	7 (10.94)	13 (20.31)		
No	57 (89.06)	52 (79.69)		

Abdominal pain			2.087	0.149
Yes	14 (21.88)	8 (12.5)		
No	50 (78.12)	57 (87.5)		

Abdominal distension			0.168	0.718
Yes	4 (6.25)	3 (4.69)		
No	60 (93.75)	62 (95.31)		

Physical examination			0.128	0.721
Yes	15 (23.44)	17 (26.56)		
No	49 (76.56)	48 (73.44)		

Change of stool habits			1.567	0.211
Yes	20 (31.25)	14 (21.88)		
No	44 (68.75)	51 (78.12)		

History of disease				
Hypertension (*n*, %)			0.611	0.434
Yes	11 (17.19)	8 (12.31)		
No	53 (82.81)	57 (87.69)		

Diabetes mellitus (*n*, %)			2.990	0.084
Yes	10 (15.63)	4 (6.15)		
No	54 (84.37)	61 (93.85)		

Heart disease (*n*, %)			1.975	0.160
Yes	10 (15.63)	5 (7.69)		
No	54 (84.37)	60 (92.31)		

Previous colonoscopy (*n*, %)			0.067	0.795
Yes	33 (51.56)	35 (53.85)		
No	31 (48.44)	30 (46.15)		

SD, standard deviation; BMI, body mass index.

**Table 2:** Efficacy of bowel cleansing in patients undergoing colonoscopy.

Parameter	0.75 L + 1.5 L group (*n* = 64)	1.5 L + 0.75 L group (n = 65)	*T*/*χ*^2^	*P* value
BBPS score (Mean ± SD)				
Rectum and sigmoid colon	2.23 ± 0.70	2.54 ± 0.56	−2.780	0.006
Transverse colon	2.17 ± 0.70	2.32 ± 0.53	−1.373	0.172
Right colon	1.94 ± 0.68	2.12 ± 0.41	−1.824	0.071
Overall colon	6.34 ± 1.88	6.98 ± 1.02	−2.408	0.017

Adequate bowel preparation *n* (%)			6.270	0.012
Yes	52 (81.25)	62 (95.38)		
No	12 (18.75)	3 (4.62)		

**Table 3:** Tolerability of bowel preparation in patients undergoing colonoscopy.

Parameter	0.75 L + 1.5 L group (*n* = 64)	1.5 L + 0.75 L group (*n* = 65)	*χ* ^2^	*P* value
Thirst *n* (%)			1.623	0.203
Yes	19 (29.69)	13 (20.00)		
No	45 (70.31)	52 (80.00)		

Nausea *n* (%)			4.481	0.034
Yes	27 (42.19)	16 (24.62)		
No	37 (57.81)	49 (75.38)		

Vomiting *n* (%)			4.458	0.035
Yes	16 (25.00)	7 (10.77)		
No	48 (75.00)	58 (89.23)		

Abdominal pain *n* (%)			0.458	0.499
Yes	9 (14.06)	12 (18.46)		
No	55 (85.94)	53 (81.54)		

Abdominal distension n (%)			0.114	0.736
Yes	17 (26.56)	19 (29.23)		
No	47 (73.44)	46 (70.77)		

Fatigue *n* (%)			6.638	0.010
Yes	14 (21.88)	4 (6.15)		
No	50 (78.12)	61 (93.85)		

Dizziness/headache *n* (%)			4.278	0.039
Yes	13 (20.31)	5 (7.69)		
No	51 (79.69)	60 (92.31)		

Anal discomfort *n* (%)			1.959	0.162
Yes	15 (23.44)	9 (13.85)		
No	49 (76.56)	56 (86.15)		

Sleep disturbance *n* (%)			2.023	0.155
Yes	18 (28.13)	26 (40.00)		
No	46 (71.87)	39 (60.00)		

## Data Availability

Data are available on request from the authors.

## Supplementary Materials

Supplementary Materials Supplementary table 1: satisfaction of bowel preparation in patients undergoing colonoscopy. Supplementary table 2: the detection rate of bowel lesions in patients undergoing colonoscopy. Supplementary table 3: correlation between bowel cleansing and lesions detection rate. Supplementary table 4: correlation between bowel cleansing and incidence of adverse reactions.

## References

[1] Jiang D., Zhang L., Liu W. (2021). Trends in cancer mortality in China from 2004 to 2018: a nationwide longitudinal study. *Cancer Communications*.

[2] Cai Z., Liu Q. (2021). Understanding the global cancer statistics 2018: implications for cancer control. *Science China Life Sciences*.

[3] Clinton S. K., Giovannucci E. L., Hursting S. D. (2020). The world cancer research fund/American institute for cancer research third expert report on diet, nutrition, physical activity, and cancer: impact and future directions. *Journal of Nutrition*.

[4] Corley D. A., Jensen C. D., Marks A. R. (2014). Adenoma detection rate and risk of colorectal cancer and death. *New England Journal of Medicine*.

[5] Dubé C., Yakubu M., McCurdy B. R. (2017). Risk of advanced adenoma, colorectal cancer, and colorectal cancer mortality in people with low-risk adenomas at baseline colonoscopy: a systematic review and meta-analysis. *American Journal of Gastroenterology*.

[6] Lebwohl B., Kastrinos F., Glick M., Rosenbaum A. J., Wang T., Neugut A. I. (2011). The impact of suboptimal bowel preparation on adenoma miss rates and the factors associated with early repeat colonoscopy. *Gastrointestinal Endoscopy*.

[7] Chokshi R. V., Hovis C. E., Hollander T., Early D. S., Wang J. S. (2012). Prevalence of missed adenomas in patients with inadequate bowel preparation on screening colonoscopy. *Gastrointestinal Endoscopy*.

[8] Rex D. K., Schoenfeld P. S., Cohen J. (2015). Quality indicators for colonoscopy. *American Journal of Gastroenterology*.

[9] Tian H., Fan Y., Yang L. (2022). The efficacy of senna bowel preparation for colonoscopy: a systematic review and meta-analysis. *Gastroenterology Nursing: the Official Journal of the Society of Gastroenterology Nurses and Associates*.

[10] Hassan C., East J., Radaelli F. (2019). Bowel preparation for colonoscopy: European society of gastrointestinal endoscopy (ESGE) guideline–update 2019. *Endoscopy*.

[11] Johnson D. A., Barkun A. N., Cohen L. B. (2014). Optimizing adequacy of bowel cleansing for colonoscopy: recommendations from the USA multi-society task force on colorectal cancer. *Gastroenterology*.

[12] Wexner S. D., Beck D. E., Baron T. H. (2006). A consensus document on bowel preparation before colonoscopy: prepared by a task force from the American society of colon and rectal surgeons (ASCRS), the American society for gastrointestinal endoscopy (ASGE), and the society of American gastrointestinal and endoscopic surgeons (SAGES). *Surgical Endoscopy*.

[13] Ell C., Fischbach W., Bronisch H. J. (2008). Randomized trial of low-volume PEG solution versus standard PEG+ electrolytes for bowel cleansing before colonoscopy. *American Journal of Gastroenterology*.

[14] Bitoun A., Ponchon T., Barthet M., Coffin B., Dugue C., Halphen M. (2006). Results of a prospective randomised multicentre controlled trial comparing a new 2-L ascorbic acid plus polyethylene glycol and electrolyte solution vs. sodium phosphate solution in patients undergoing elective colonoscopy. *Alimentary Pharmacology & Therapeutics*.

[15] Ponchon T., Boustière C., Heresbach D., Hagege H., Tarrerias A. L., Halphen M. (2013). A low-volume polyethylene glycol plus ascorbate solution for bowel cleansing prior to colonoscopy: the NORMO randomised clinical trial. *Digestive and Liver Disease*.

[16] DiPalma J. A., Wolff B. G., Meagher A., Cleveland M. (2003). Comparison of reduced volume versus four liters sulfate-free electrolyte lavage solutions for colonoscopy colon cleansing. *American Journal of Gastroenterology*.

[17] Rodriguez De Miguel C., Serradesanferm A., Del Manzano S. (2012). Timing of polyethylene glycol administration is a key factor in the tolerability and efficacy of colon preparation in colorectal cancer screening. *Gastroenterología y Hepatología*.

[18] Park S. S., Sinn D. H., Kim Y. H. (2010). Efficacy and tolerability of split-dose magnesium citrate: low-volume (2 liters) polyethylene glycol vs. single- or split-dose polyethylene glycol bowel preparation for morning colonoscopy. *American Journal of Gastroenterology*.

[19] Rodríguez de Miguel C., Serradesanferm A., López-Cerón M. (2015). Ascorbic acid PEG-2L is superior for early morning colonoscopies in colorectal cancer screening programs: a prospective non-randomized controlled trial. *Gastroenterología y Hepatología*.

[20] Huh C. W., Gweon T. G., Seo M., Ji J. S., Kim B. W., Choi H. (2018). Validation of same-day bowel preparation regimen using 4 L polyethylene glycol: comparison of morning and afternoon colonoscopy. *Medicine (Baltimore)*.

[21] Lawrance I. C., Willert R. P., Murray K. (2013). A validated bowel-preparation tolerability questionnaire and assessment of three commonly used bowel-cleansing agents. *Digestive Diseases and Sciences*.

[22] Hatoum H. T., Lin S. J., Joseph R. E., Dahdal D. N. (2016). Validation of a patient satisfaction scale in patients undergoing bowel preparation prior to colonoscopy. *The Patient-Patient-Centered Outcomes Research*.

[23] Matro R., Shnitser A., Spodik M. (2010). Efficacy of morning-only compared with split-dose polyethylene glycol electrolyte solution for afternoon colonoscopy: a randomized controlled single-blind study. *American Journal of Gastroenterology*.

[24] Varughese S., Kumar A. R., George A., Castro F. J. (2010). Morning-only one-gallon polyethylene glycol improves bowel cleansing for afternoon colonoscopies: a randomized endoscopist-blinded prospective study. *American Journal of Gastroenterology*.

[25] Mohamed R., Hilsden R. J., Dube C., Rostom A. (2016). Split-dose polyethylene glycol is superior to single dose for colonoscopy preparation: results of a randomized controlled trial. *Canadian Journal of Gastroenterology and Hepatology*.

[26] Rutter M. D. (2014). Importance of nonpolypoid (flat and depressed) colorectal neoplasms in screening for CRC in patients with IBD. *Gastrointestinal Endoscopy Clinics of North America*.

[27] Klinger A. L., Kann B. R. (2019). Endoscopy in inflammatory bowel disease. *Surgical Clinics of North America*.

[28] Banerjee R., Pal P. (2021). Endoscopic evaluation and therapeutic considerations of small bowel crohn’s disease. *Gastroenterology Insights*.

[29] Briot C., Faure P., Parmentier A. L. (2019). Efficacy, tolerability, and safety of low-volume bowel preparations for patients with inflammatory bowel diseases: the French multicentre CLEAN study. *Journal of Crohn’s and Colitis*.

[30] Negreanu L., Voiosu T., State M., Mateescu R. B (2020). Quality of colonoscopy preparation in patients with inflammatory bowel disease: retrospective analysis of 348 colonoscopies. *Journal of International Medical Research*.

[31] Kotwal V. S., Attar B. M., Carballo M. D. (2014). Morning-only polyethylene glycol is non-inferior but less preferred by hospitalized patients as compared with split-dose bowel preparation. *Journal of Clinical Gastroenterology*.

[32] Zhang S., Li M., Zhao Y. (2015). 3-L split-dose is superior to 2-L polyethylene glycol in bowel cleansing in Chinese population: a multicenter randomized, controlled trial. *Medicine (Baltimore)*.

[33] Pagnini C., Di Paolo M. C., Mariani B. M. (2021). Mayo endoscopic score and ulcerative colitis endoscopic index are equally effective for endoscopic activity evaluation in ulcerative colitis patients in a real life setting. *Gastroenterology Insights*.

