# Supplementary material for: Efficacy and Tolerability of Two Different Low-Volume Split-Dose Polyethylene Glycol Electrolytes Solution Bowel Preparation for Morning Colonoscopy
Source: Can J Gastroenterol Hepatol. 2022 Aug 31;2022:8169649. doi: 10.1155/2022/8169649 (PMC9453094; doi:10.1155/2022/8169649)
Supplement: Supplementary Materials — Supplementary table 1: satisfaction of bowel preparation in patients undergoing colonoscopy. Supplementary table 2: the detection rate of bowel lesions in patients undergoing colonoscopy. Supplementary table 3: correlation between bowel cleansing and lesions detection rate. Supplementary table 4: correlation between bowel cleansing and incidence of adverse reactions. [file 8169649.f1.zip › Supplementary table 1.doc]

| **Supplementary table 1. Satisfaction of bowel preparation in patients undergoing colonoscopy** | | | | | |  |
| --- | --- | --- | --- | --- | --- | --- |
| **Parameter** | | **0.75L+1.5L group（*n*=64）** | **1.5L+0.75L group**  **（*n*=65）** | ***χ^2^*** | ***P* value** | |
| Easy or difficult to consume the drug *n*（%） | |  |  | 0.081 | 0.826 | |
| Very easy | | 7（10.94） | 7（10.77） |  |  | |
| Easy | | 19（29.69） | 18（27.69） |  |  | |
| Tolerable | | 32（50.00） | 37（56.92） |  |  | |
| Difficult | | 5（7.81） | 3（4.62） |  |  | |
| Very difficult | | 1（1.56） | 0（0.00） |  |  | |
| The taste of the drug *n*（%） | |  |  | 0.122 | 0.805 | |
| Very satisfied | | 1（1.56） | 3（4.62） |  |  | |
| Satisfied | | 17（26.56） | 17（26.15） |  |  | |
| General | | 37（57.82） | 35（53.85） |  |  | |
| Dissatisfied | | 9（14.06） | 10（15.38） |  |  | |
| Very dissatisfied | | 0（0.00） | 0（0.00） |  |  | |
| The flavor of the drug *n*（%） | |  |  | 0.350 | 0.603 | |
| Very satisfied | | 5（7.81） | 4（6.15） |  |  | |
| Satisfied | | 22（34.38） | 25（38.46） |  |  | |
| General | | 33（51.56） | 36（55.39） |  |  | |
| Dissatisfied | | 4（6.25） | 0（0.00） |  |  | |
| Very dissatisfied | | 0（0.00） | 0（0.00） |  |  | |
| The dose of the drug *n*（%） | |  |  | 0.059 | 0.822 | |
| Very satisfied | | 2（3.12） | 2（3.08） |  |  | |
| Satisfied | | 19（29.69） | 18（27.69） |  |  | |
| General | | 30（46.88） | 31（47.69） |  |  | |
| Dissatisfied | | 13（20.31） | 14（21.54） |  |  | |
| Very dissatisfied | | 0（0.00） | 0（0.00） |  |  | |
| The regimen for bowel preparation *n*（%） |  | |  | 6.480 | 0.011 |  |
| Very satisfied | | 7（10.94） | 15（23.08） |  |  |  |
| Satisfied | | 34（53.13） | 37（56.92） |  |  |  |
| General | | 21（32.81） | 13（0.20） |  |  |  |
| Dissatisfied | | 2（3.12） | 0（0.00） |  |  |  |
| Very dissatisfied | | 0（0.00） | 0（0.00） |  |  |  |
| Willingness to repeat bowel preparation  *n*（%） |  | |  | 5.886 | 0.015 |  |
| Yes | | 45（70.31） | 57（87.69） |  |  |  |
| No | | 19（29.69） | 8（12.31） |  |  |  |
