# Supplementary material for: Efficacy and Tolerability of Two Different Low-Volume Split-Dose Polyethylene Glycol Electrolytes Solution Bowel Preparation for Morning Colonoscopy
Source: Can J Gastroenterol Hepatol. 2022 Aug 31;2022:8169649. doi: 10.1155/2022/8169649 (PMC9453094; doi:10.1155/2022/8169649)
Supplement: Supplementary Materials — Supplementary table 1: satisfaction of bowel preparation in patients undergoing colonoscopy. Supplementary table 2: the detection rate of bowel lesions in patients undergoing colonoscopy. Supplementary table 3: correlation between bowel cleansing and lesions detection rate. Supplementary table 4: correlation between bowel cleansing and incidence of adverse reactions. [file 8169649.f1.zip › Supplementary table 2.doc]

| **Supplementary table 2. The detection rate of bowel lesions in patients undergoing colonoscopy** | | | | | | |
| --- | --- | --- | --- | --- | --- | --- |
| **Parameter** | | **0.75L+1.5L group（*n*=64）** | **1.5L+0.75L group**  **（*n*=65）** | | ***χ^2^*** | ***P* value** |
| Polyps *n*（%） |  | | |  | 0.394 | 0.530 |
| Yes | | 22（34.38） | | 19（29.23） |  |  |
| No | | 42（65.62） | | 46（70.77） |  |  |
| Adenoma *n*（%） | |  | |  | 1.825 | 0.365 |
| Yes | | 1（1.56） | | 4（6.15） |  |  |
| No | | 63（98.44） | | 61（93.85） |  |  |
| Inflammation *n*（%） | |  | |  | 0.183 | 0.668 |
| Yes | | 10（15.63） | | 12（18.46） |  |  |
| No | | 54（84.37） | | 53（81.54） |  |  |
| Suspected cancer *n*（%） | |  | |  | 1.024 | 0.496 |
| Yes | | 1（1.56） | | 0（0） |  |  |
| No | | 63（98.44） | | 65（100） |  |  |
| Other lesions n（%） | |  | |  | 0.326 | 1.000 |
| Yes | | 1（1.56） | | 2（3.08） |  |  |
| No | | 63（98.44） | | 63（96.92） |  |  |
| Total lesions | |  | |  | 0.063 | 0.802 |
| Yes | | 26（40.62） | | 25（38.46） |  |  |
| No | | 38（59.38） | | 40（61.54） |  |  |
