# Supplementary material for: Efficacy and Tolerability of Two Different Low-Volume Split-Dose Polyethylene Glycol Electrolytes Solution Bowel Preparation for Morning Colonoscopy
Source: Can J Gastroenterol Hepatol. 2022 Aug 31;2022:8169649. doi: 10.1155/2022/8169649 (PMC9453094; doi:10.1155/2022/8169649)
Supplement: Supplementary Materials — Supplementary table 1: satisfaction of bowel preparation in patients undergoing colonoscopy. Supplementary table 2: the detection rate of bowel lesions in patients undergoing colonoscopy. Supplementary table 3: correlation between bowel cleansing and lesions detection rate. Supplementary table 4: correlation between bowel cleansing and incidence of adverse reactions. [file 8169649.f1.zip › Supplementary table 3.doc]

| **Supplementary table 3. Correlation between bowel cleansing and lesions detection rate** | | | | | |
| --- | --- | --- | --- | --- | --- |
| **Group** | **Bowel cleansing** | **Detection of lesions** | | ***χ^2^*** | ***P* value** |
|  |  | **Yes** | **No** |  |  |
| 0.75L+1.5L （*n*=64） | ≥ 6 scores | 19 | 33 | 1.920 | 0.202 |
|  | < 6 scores | 7 | 5 |  |  |
| 1.5L+0.75L （*n*=65） | ≥ 6 scores | 33 | 29 | 0.454 | 0.602 |
|  | < 6 scores | 1 | 2 |  |  |
